# Characterization of mesenchymal stem cells derived from the equine synovial fluid and membrane

**DOI:** 10.1186/s12917-015-0531-5

**Published:** 2015-11-10

**Authors:** Aline Ambrogi Franco Prado, Phelipe Oliveira Favaron, Luis Claudio Lopes Correia da Silva, Raquel Yvonne Arantes Baccarin, Maria Angelica Miglino, Durvanei Augusto Maria

**Affiliations:** Department of Surgery, School of Veterinary Medicine and Animal Science, University of Sao Paulo, Av. Prof. Dr. Orlando Marques de Paiva, 87, Cidade Universitária, 05508-270 São Paulo, SP Brazil; Laboratory of Biochemical and Biophisic, Butantan Institute, Av. Dr. Vital Brasil, 1500, 05503-900 Sao Paulo, Brazil

**Keywords:** Stem cells, Synovial Fluid, Horse, Regenerative medicine

## Abstract

**Background:**

Isolation of mesenchymal stem cells (MSCs) in equines, has been reported for different tissues including bone marrow, adipose, umbilical cord, peripheral blood, and yolk sac. In regard to the MSCs derived from synovial fluid (SF) or membrane (SM), there is data available for humans, dogs, pigs, goats and horses. Especially in equines, these cells have being considered promising candidates for articular regeneration. Herein, we established and characterized MSCs obtained from equine SF and SM. Samples were obtained during arthroscopy and cultured using MEM (Minimum Essential Medium). MSCs were characterized by morphology and expression of specific markers for stem cells, pluripotency, inflammation, and cell cycle.

**Results:**

The medium MEM was more effective (97 % ± 2) to maintain both cultures. The cultures were composed by adherent cells with fibroblast-like shape, which had a growth pattern represented by a sigmoidal curve. After the expansion, the cells were analyzed by flow cytometry for stem cells, inflammatory, and cell cycle markers, and both lineages showed significant expression of CD45, Oct3/4, Nanog, CD105, CD90, CD34, CD117, CD133, TRA-1-81, VEGF, and LY6a. In contrast, there were differences in the cell cycle phases between the lineages, which was not observed in relation to the mitochondrial electrical potential.

**Conclusion:**

Given the large impact that joint pathology has on the athletic performance horses, our results suggested that the SF and SM are promising sources of stem cells with satisfactory characteristics of growth and gene expression that can be used in equine regenerative medicine.

## Background

Joint destruction, a consequence of the development of inflammatory, degenerative, and rheumatic diseases, leads to severe disabilities [1]. Its high prevalence and impact on the ability to work, coupled with the high cost of therapeutic procedures, makes it an important problem for veterinary clinics. Horses that are intensely active, and also have an especially high level of performance, are susceptible to musculoskeletal disorders, which can culminate in low athletic performance and pathologies [2]. These injuries require a strategic clinical application to restore the cartilage, bone, tendon, and muscles [3]. The synovial fluid produced by the synovial membrane is viscous, light yellow, clear, and free of particulate matter, fibrinogen and other coagulation factors [4]. Despite being treated as an inert material for decades, the articular cartilage is a tissue in constant regeneration. In addition, data has shown that the synovial membrane and fluid are sources of mesenchymal progenitor cells, which support the ability of *in vivo* cartilage repair [5]. Mesenchymal stem cells (MSCs) can be defined as a population of adherent cells, fibroblastic in shape, and multipotent with high proliferative abilities. Besides the first stem cells were obtained from the bone marrow, the continued search for new sources of stem cells coupled with technological advances in cell isolation, has allowed for the identification of mesenchymal stem cells from several adult tissues, such as periosteum, musculoskeletal tissue, adipose, and the synovial membrane and fluid [6]. Although bone marrow is considered a good and acceptable source of stem cells, the synovial membrane and its fluid are tissue-specific, which leads to a chondrogenic and expansion potential greater than other sources. Furthermore, these cells can be obtained by minimally invasive techniques [6–9]. Previously data demonstrated the multipotency of stromal cells obtained from the synovial fluid of horses with intraarticular injury and synovitis [10]. The synovial fluid-derived MSCs expressed CD90, CD105, CD44, CD11a/CD18, and MHC class I and II. In addition, *in vitro* the cells were able to differentiate in osteogenic, adipogenic, chondrogenic, and tenogenic lineages [10]. Considering that treating osteoarthritis, which causes persistent pain and contributes to chronic lameness, is difficult in chronic diseases, with a reserved prognosis [11–13], and the growing interest for this field especially in regard to the search for new strategies for treatment, we are establishing a protocol to culture and characterize mesenchymal stem cells not only from equine synovial fluid but also from the synovial membrane, which in the future can be used to treat osteoarthritis, especially when surgical intervention is not viable.

## Methods

### Sampling and cell culture

This research was approved by the Bioethics Committee from the School of Veterinary Medicine and Animal Science, University of Sao Paulo, Sao Paulo, Brazil (Protocol 2771/2012). Synovial fluid and membrane were obtained from the tibiotarsal and metacarpophalangeal joints during arthroscopic procedure in ten horses with osteochondrosis, which were included in the research after agreement of the owners. Samples were collected in a sterile syringe and transferred to tissue culture flasks (Corning, NY, USA) with 5 ml of culture medium MEM (Minimum Essential Medium—GIBCO^™^), supplemented with 10 % of fetal bovine serum (FBS) and 1 % of penicillin and streptomycin. Culture flasks were incubated at 37 °C with a relative humidity atmosphere of 5 % CO_2_. After 24 and 48 h, non-adherent cells were removed and the medium was replaced. Every 3 days, 70 % of the medium was replaced and at an 80 % confluence, the cells were enzymatically dissociated using 0.25 % trypsin (Invitrogen, Carlsbad, CA, USA) for 5 min at 37 °C. Thereafter, the cells were centrifuged at 1000 rpm for 5 min and the pellet that resulted was resuspended in 1 ml of a culture medium and transferred to culture flasks. The growth and morphology of the adherent cells were followed by photo documentation in an inverted microscopy (NIKON ECLIPSE TS-100), coupled with an image system (CCD—Sony). For freezing, cryotubes with 1 × 10^4^ cells and freezing medium (90 % of FBS and 10 % of DMSO) were maintained in liquid nitrogen.

### Growth curve

The growth curve was performed in order to evaluate the expansion and replication abilities, standardize the optimal cell concentration for cell growth, and assess their kinetic behavior. After initially establishing the culture, samples were obtained during the periods of 24, 48, 72, 96, 120, 144, and 168 h. The evaluation of the cell number and viability were performed in triplicate by cell counting in the Neubauer chamber using Trypan Blue 0.2 % (v:v) staining.

### Immunophenotyping by flow cytometry

Adherent cells on passage 7 were resuspended in FACS (fluorescence-activated cell sorting) buffer, and the concentration adjusted to 10^5^cells/mL. For intracytoplasmic and nuclear markers, cells were permeabilized with 5 μl 0.1 % Triton X-100 for 30 min prior to incubation with primary antibodies (concentration of 1:100) specific for stem cells, inflammation, and cell cycle progression: Oct 3/4 (C-10, SC-5279), Nanog (n-17, SC-30331), CD45/OX1 (SC-53045), CD105 (2Q1707, SC-71042), CD90 (Thy-1, aTHy-1A1), CD34 (BI-3C5, SC: 19621), Caspase-3 (SC-7272), HSP-47 (SC-8352), P21 (SC-6246), Ki67 (Ab – 15580), Cyclin-D1 (AB-27618), P53 (Ab −26), TRA-1-81 (SC-21706), MCP-1 (SC- 32771), TNF-R1 (SC, 52746), all from Santa Cruz Biotechnology, as well as CD117 (c-Kit, SCF-Receptor Ab-6, RB-1518-R7, Thermo Scientific, Lab Vision Corporation, Fremont, CA, USA), VEGF-R1 (Clone VG1, M7273, DakoCytomation, CA, USA), COX-2 (Cayman Chemical Co, EUA), CD11b (MCA-551FT), Ly6a (Ab – 51317), CD1a (SC-18885), and CD133 (Mab4310, Merck Millipore), all for 45 min at room temperature. Then, cells were incubated for 2 h with a secondary antibody (Anti-Mouse FITC, DakoCytomation, Santa Cruz Biotechnology). The analysis was performed using a flow cytometer (FACSCalibur, BD). The expression of surface markers was determined by comparison with an isotype control analyzed by a histogram on the CELLQUEST program.

### Analysis of the cell cycle

For the evaluation of the cell cycle, cells were resuspended in FACS flow buffer and centrifuged for 3 min at 2000 rpm. The supernatant was discarded and the cells were carefully resuspended in 1 ml of 70 % RNAse ethanol, transferred to microtubes and stored at −20 °C. Then, samples were centrifuged again at 2000 rpm for 5 min and the cells were resuspended in 1 ml of cytometry buffer. After centrifugation, cells were resuspended in a propidium iodide solution, which was prepared using 5 ml of PBS (phosphate buffer solution), 5 μl of Triton 100 (0,01 % v/v), 50 μl of RNAse A (2 mg/ml) and 20 μl of propidium iodide (5 mg/ml). The analysis was performed using a FACSCalibur flow cytometry and the Win 2.8 MDI program.

### Analysis of the electrical potential of the mitochondrial membrane

Rhodamine 123 (Invitrogen, EUA) was used for the analysis of mitochondrial membrane potential (Δψm). Cells from the synovial fluid and the membrane were trypsinized and centrifuged at 1500 rpm for 10 min, the supernatant was discarded and 5 μl of Rhodamine 123 (5 mg/ml) was added. Then, the cells were incubated in CO_2_ (5 %) at 37 °C for 30 min. After this period, the cells were centrifuged, the supernatant was discarded, and the precipitate was resuspended in 100 μl of buffer for FACsFlow cytometry (BD). The analysis was performed on a FACSCalibur flow cytometry (BD) and analyzed by the Win MDI 2.8 program.

### Statistical analysis

The statistical analysis was performed using the ANOVA test, followed by a multiple comparison test, TUKEY-KRAMER. All values were expressed as mean ± standard deviation (SD), considering as critical, the level for significance values *p* < 0.05. Significant differences between the groups were indicated by: ***p < 0.001; ** *p* < 0.01; * *p* < 0.05. To perform the statistical analysis, the GraphPad Prism version 5.0 was used.

## Results

### Morphology and culture growth

**Fig. 1:**
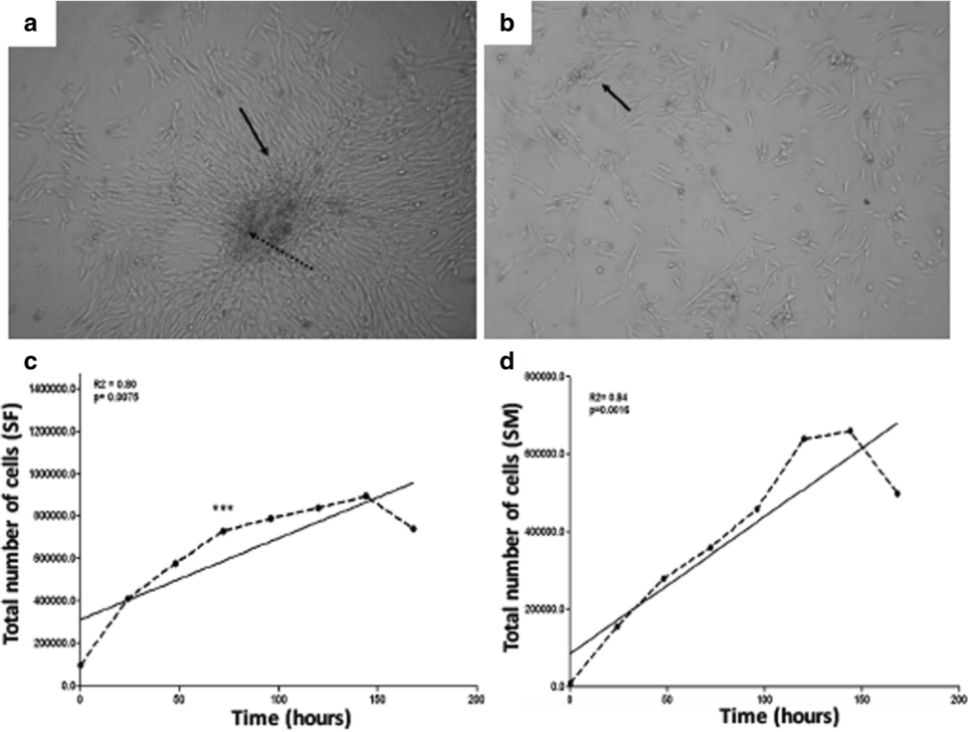
Cell morphology and growth. **a** and (**b**): Both cells, from the synovial fluid and the membrane, respectively, had a fibroblast-like appearance with a fusiform shape, growing in colonies. **c** and **d**: Both cultures had a growth pattern represented by a sigmoidal curve. The synovial fluid cells (SF) showed a period of adjustment without proliferation (lag phase, approximately 50 h of culture). Exponential growth was observed until 150 h, and after 168 h a decline phase was observed (**c**). In contrast for synovial membrane cells (SM) the lag phase was reached approximately 24 h of culture, followed by a exponential growth until 150 h and a decline phase close to 160 h (**d**)

In order to standardize the best culture medium to maintain the synovial fluid and membrane, 4 tests were performed using 4 different types of mediums supplemented with 10 % of FBS and 1 % of antibiotics - penicillin and streptomycin (Table 1). The mediums MEM (Minimum Essential Medium) and DMEM-H (Dulbecco Modified Essential Medium High Glucose) showed the best results and were effective in maintaining the cells with satisfactory characteristics of growth and in an undifferentiated stage. As MEM was more effective (97 % ±2), this medium was chosen to culture the cells and perform the experiments. Cells cultivated in mediums RPMI-1640 (Roswell Park Memorial Institute) and DMEM/F12 (Dulbecco Modified Eagle Medium) showed unsatisfactory abilities for proliferation and growth. However, in the culture, the cells also showed a fibroblast-like morphology. Nevertheless, after the culture expansion, a loss of cell adhesion in the substrate and development of multi-cell aggregates in the culture, resulting in cell death, was observed (Table 1). Both cells from the synovial fluid and the membrane had a fibroblast-like appearance with a fusiform shape (Fig. 1a and b) that was maintained after freezing. Cells were cultivated until the 11th passage and showed the same morphology during this period. Both, the synovial fluid and membrane cells had a growth pattern represented by a sigmoidal curve that reflected the phases of cell adaptation under growing conditions, availability of nutrients in the medium, and support for cell adhesion. The synovial fluid cells showed a period of adjustment in which proliferation does not occur, especially after the addition of the cells to the culture medium. At this stage there was an intense metabolic activity that was dependent on density and cell adhesion. It corresponded to the lag phase, with approximately 50 h of culture. During the exponential growth phase, cell proliferation was maximal and constant, occurring until 150 h of culture. The stationary phase, or plateau, when the growth rate decreased, was not determined in these culture conditions. However, cell death progressively increased and cells detached from the substrate. After 168 h, there was a decline phase or cell death, which showed a drastic decrease in density and cell viability. The dose curve, time and cell density showed a significant positive correlation, with r^2^ of 0.80 (Fig. 1c). In relation to the growth of the synovial membrane cells, the lag phase occurred after approximately 24 h of culture. The exponential growth curve occurred from 50 to 150 h. In our culture condition, the stationary phase also was not determined for synovial membrane cells. In contrast, cell death and detachment of the cells from the substrate progressively increased, as demonstrated for synovial fluid cells. Finally, the decline phase occurred close to 160 h, and the reduction corresponded to 68 %. The dose curve, time and cell density showed a significant positive correlation, with r^2^ of 0.84 (Fig. 1d).

**Table 1:** Culture media used to isolate the mesenchymal stem cells derived from the equine synovial fluid and membrane

Medium	Results
DMEM-High Glucose (LGC Biotecnologia, Cotia, Sao Paulo, Brazil), 10 % FBS (fetal bovine serum) characterized (HyClone, Thermo Scientific), 1 % antibiotic solution (Penicilin G 10.00U mL, 25 mg mL, Streptomycin 10.000 mg mL), 1 % l-glutamine 200 Mm, and 1 % non-essential amino acids. All from Invitrogen, Carlsbad, CA, USA	Quickly growth. Cells with fibroblast-like morphology. Cell adhesion on plates. 80 ± 12 % of efficiency
MEM (LGC Biotecnologia, Cotia, Sao Paulo, Brazil), 10 % FBS characterized (HyClone, Thermo Scientific), 1 % antibiotic solution (Penicilin G 10.00U mL, 25 mg mL, Streptomycin 10.000 mg mL), 1 % l-glutamine 200 Mm, and 1 % non-essential amino acids. All from Invitrogen, Carlsbad, CA, USA	Quickly growth. Cells with fibroblast-like morphology. Cell adhesion on plates. 97 ± 2 % of efficiency
RPMI-1640 (LGC Biotecnologia, Cotia, Sao Paulo, Brazil), 10 % FBS characterized, 1 % antibiotic solution (Penicilin G 10.00U mL, 25 mg mL, Streptomycin 10.000 mg mL), 1 % l-glutamine 200 Mm, and 1 % non-essential amino acids. All from Invitrogen, Carlsbad, CA, USA. 1 % antibiotic solution (Penicilin G 10.00U mL, 25 mg mL, Streptomycin 10.000 mg mL), 1 % l-glutamine 200 Mm, and 1 % non-essential amino acids. All from Invitrogen, Carlsbad, CA, USA	Slow growth. Cells with fibroblast-like morphology. Few adhesion on plates. 10 ± 8.3 % of efficiency
DMEM/HAM’S-F12 (1:1) (LGC Biotecnologia, Cotia, Sao Paulo, Brazil), 10 % FBS characterized, 1 % antibiotic solution (Penicilin G 10.00U mL, 25 mg mL, Streptomycin 10.000 mg mL), 1 % l-glutamine 200 Mm, and 1 % non-essential amino acids. All from Invitrogen, Carlsbad, CA, USA	Slow growth. Cells with fibroblast-like morphology. Unsatisfactory adhesion on plates. 5 ± 1.2 % of efficiency

### Immunophenotype, cell characterization, cell cycle progression and proliferation rate

**Fig. 2:**
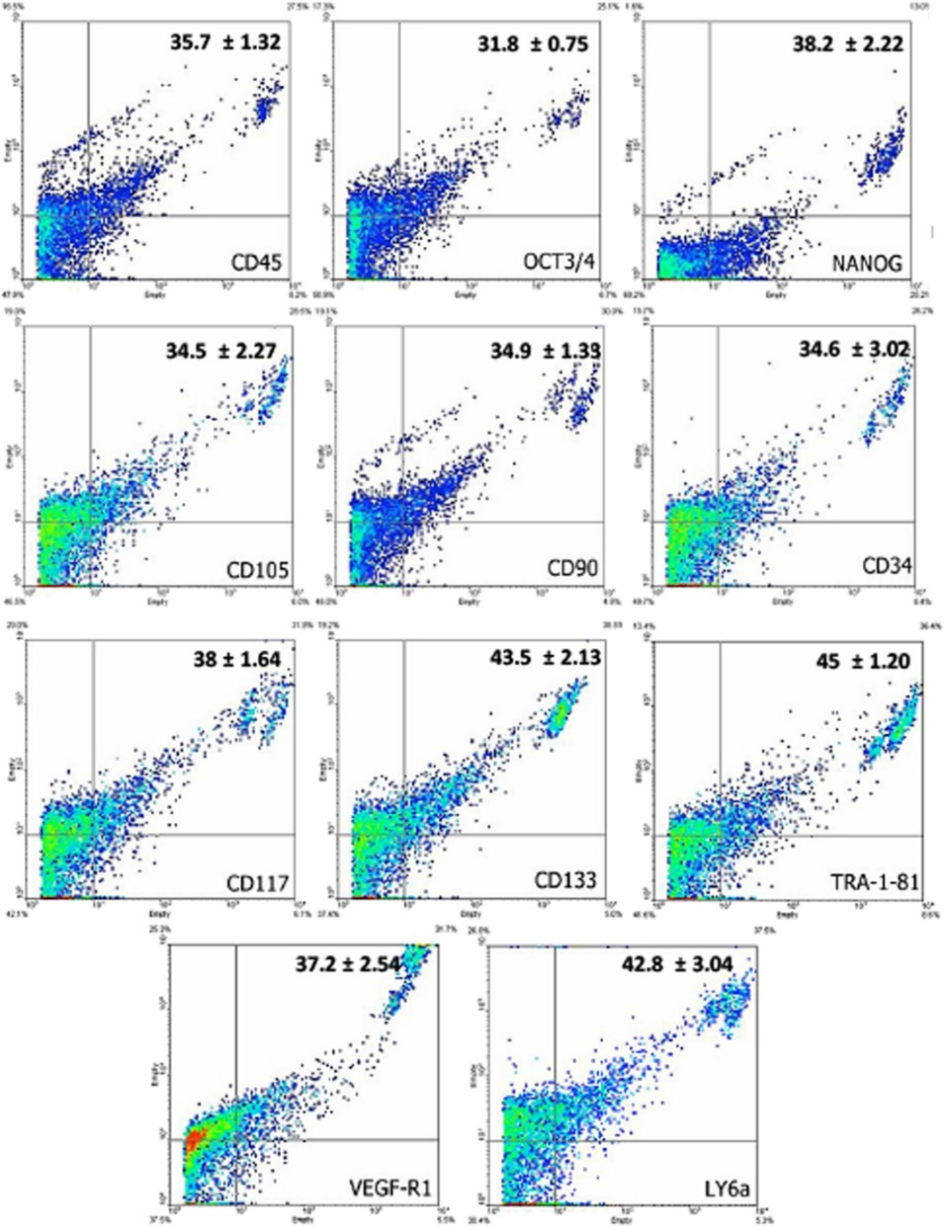
Expression of markers by flow cytometry on cells derived from the synovial fluid. The synovial fluid cells showed significant expression of hematopoietic (CD45, CD34, CD117, and CD133), mesenchymal (CD105 and CD90), pluripotency (Oct3/4 and Nanog), embryonic (Tra-1-81), and inflammatory (VEGF-R1 and LY6a) markers

**Fig. 3:**
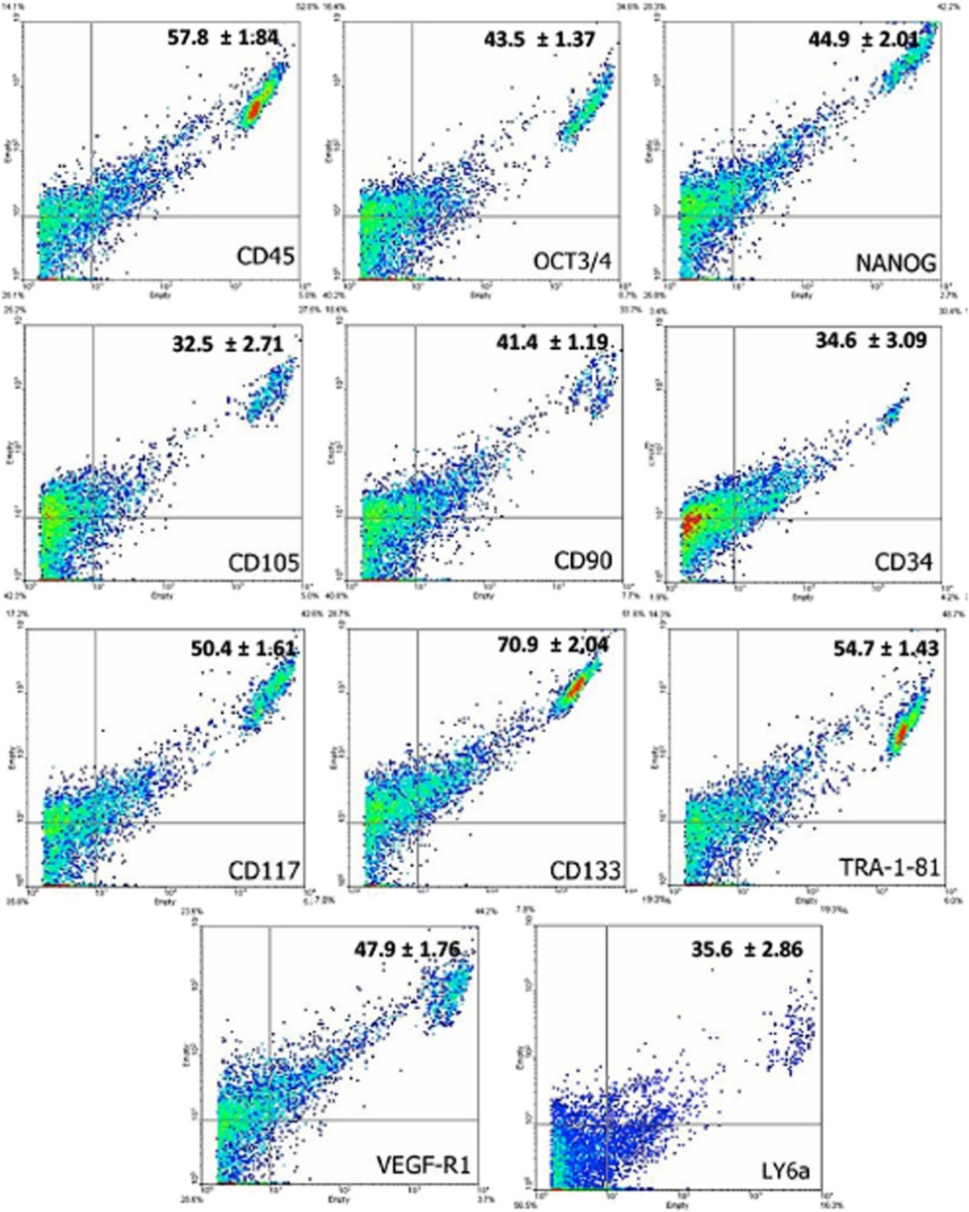
Expression of markers by flow cytometry on cells derived from the synovial membrane. The synovial membrane cells showed significant expression of hematopoietic (CD45, CD34, CD117, and CD133), mesenchymal (CD105 and CD90), pluripotency (Oct3/4 and Nanog), embryonic (Tra-1-81), and inflammatory (VEGF-R1 and LY6a) markers

**Fig. 4:**
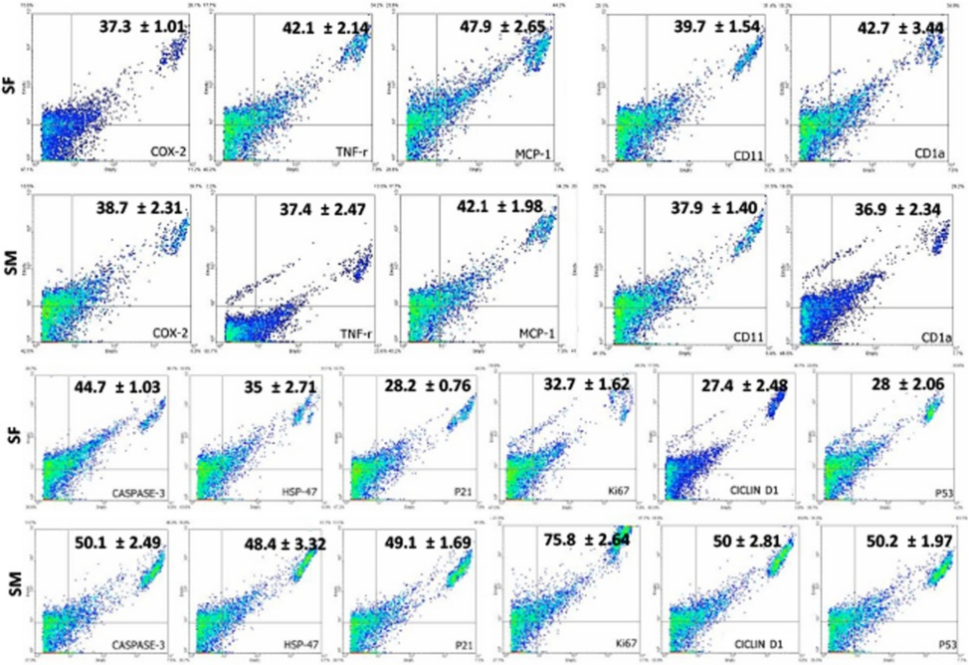
Expression of inflammatory and cell cycle progression markers by flow cytometry on cells derived from the synovial fluid and membrane. The TNF-r, MCP-1 and CD1a were significantly reduced in the synovial membrane cells compared to the synovial fluid cells. There was no difference in relation to the expression of the other markers (Cox-2 and CD11) between the two cultures and all markers showed significant expression. In relation to cell cycle progression markers, statistical difference between the cultures was observed only in relation to the expression of HSP47, which was increased in the synovial fluid cultures. The expression of other markers (Caspase-3, p21, Ki67, ciclin-D1, and p53) was similar between both cultures

**Fig. 5:**
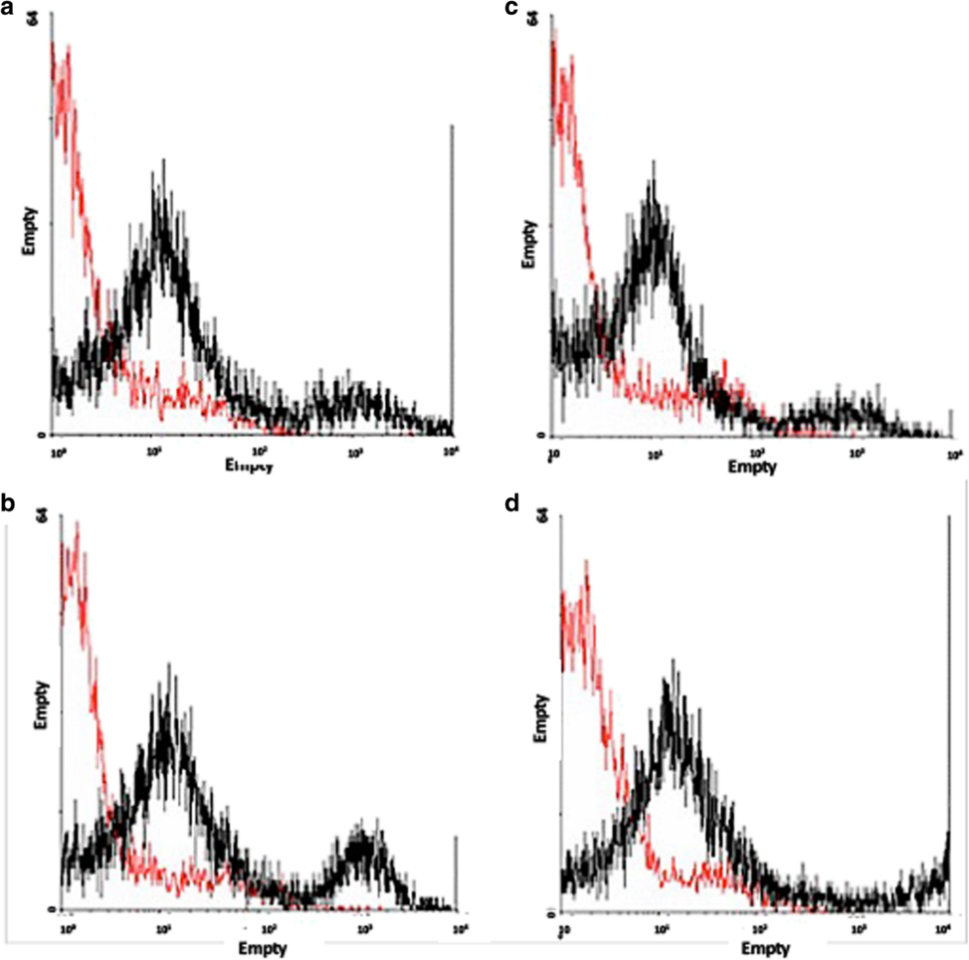
Isotype controls. **a** and (**b**) Red line corresponds to the IgG2a isotype control (mouse) and in black the specific binding in samples of synovial membrane and fluid, respectively. **c** and (**d**) Red line corresponds to the IgM isotype control (mouse) and in black the specific binding in samples of synovial membrane and fluid, respectively

**Fig. 6:**
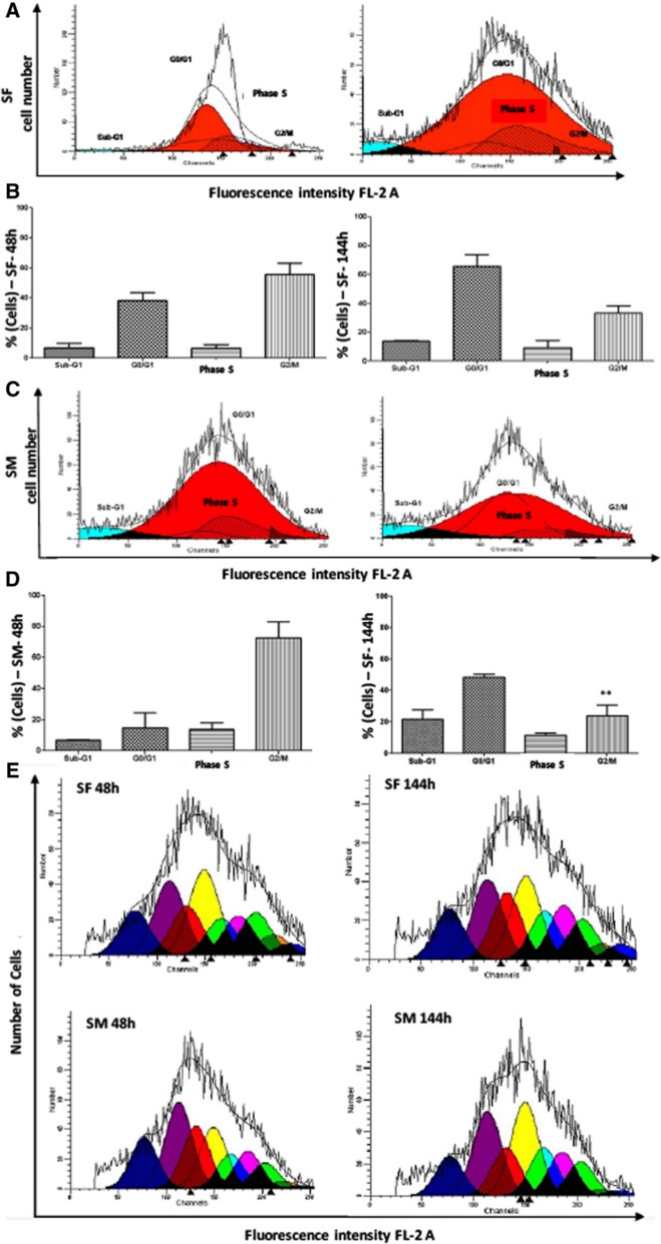
Analysis of the cell cycle on cells cultures derived from the synovial fluid and membrane. There were no significant differences in the proportions of cell distribution in cell cycle phases between the culture of synovial fluid (SF) cells of 48 and 144 h (**a** and **b**). In contrast, there were significant differences in the proportions of synovial membrane (SM) cells between 48 and 144 h. There was a decrease in the G2/M cell population, as demonstrated in the kinetics of cell growth and viability. After 144 h, the synovial membrane cells decreased their proliferative ability (**c** and **d**). **e**: The results showed a higher capacity for proliferation of synovial fluid (SF) cells between 48 and 144 h when compared to synovial membrane (SM) cells, which showed lower proliferation after 144 h in culture. The acquisitions were obtained by flow cytometer were analyzed by the Wizard Proliferation

After the expansion, the cells obtained from synovial fluid and membrane in the 7th passage were analyzed by flow cytometry for stem cells, inflammatory, and cell cycle progression markers. The cells showed a significant expression of CD45 (35.7 and 57.8 %), Oct3/4 (31.8 and 43.3 %), Nanog (38.2 and 44.9 %), CD105 (34.5 and 32.5 %), CD90 (34.9 and 41.4 %), CD34 (34.6 and 34.6 %), CD117 (38 and 50.4 %), CD133 (43.5 and 70.9 %), TRA-1-81 (45 and 54.7 %), VEGF (37.2 and 47.9 %), and LY6a (42.8 and 35.6 %), respectively. Representative dotplots of acquisition for each marker by flow cytometry, and the mean and standard deviation are shown on Figs. 2 and 3, for stem cells from the synovial fluid and membrane, respectively. Confirming the inflammatory potential of expression of the VEGF-R1 receptor in synovial fluid cells, the expression of pro-inflammatory markers was evaluated. The TNF-R1 receptor, the chemotactic protein MCP-1 and CD1a showed decreased expression in synovial membrane cells, while the other analyzed markers (COX-2 and CD11) did not show significant differences between the synovial fluid and the membrane, despite being positive in both colonies (Fig. 4). The HSP47, a collagen-specific molecular chaperone responsible for the collagen synthesis showed increased expression in the synovial fluid cultures in comparison to the membrane. Moreover, high expression of Ki-67 was observed in both cultures. The expression of other markers (Caspase-3, p21, ciclin-D1, and p53) was similar between both cultures (Fig. 4). For flow cytometry analysis, mouse IgG2 and IgM were used as isotype controls (Fig. 5). There were no significant differences in the proportions of cell distribution in cell cycle phases between the culture of synovial fluid cells of 48 and 144 h, showing the maintenance of their proliferative potential, as showed for the kinetics of cell growth and viability. Representative histograms acquired by the ModFit program of different cell cycle phases as well as the comparison of the average values, are shown on Fig. 6a and b. However, there were significant differences in the proportions of synovial membrane cells between 48 and 144 h. There was a decrease in the G2/M cell population, as demonstrated in the kinetics of cell growth and viability. After 144 h, the synovial membrane cells decreased their proliferative ability significantly. Representative histograms of cell cycle phases, as well as the average values, are shown on Fig. 6c and d. The protocol used with the CSFE demonstrated how the marker was equally divided between the daughter cells during each cell division. The results showed a higher capacity for proliferation of synovial fluid cells between 48 and 144 h when compared to synovial membrane cells, which showed lower proliferation after 144 h in culture. The acquisitions obtained by a flow cytometer were analyzed by the Wizard Proliferation (Fig. 6e).

### Analysis of mitochondrial electrical potential

**Fig. 7:**
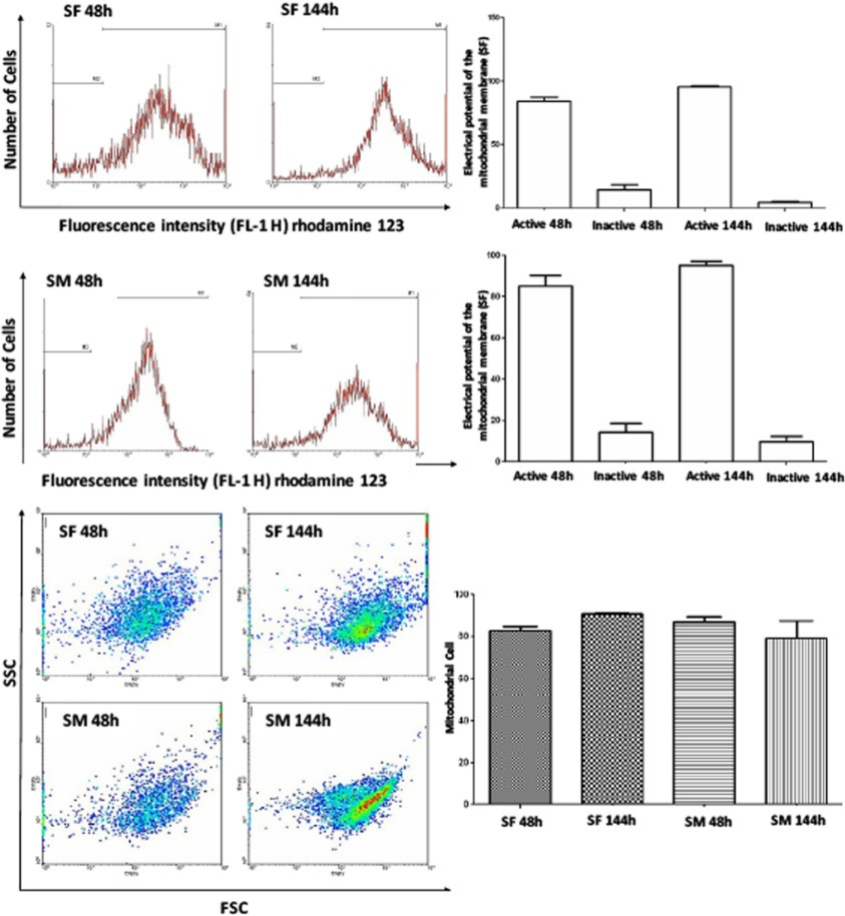
Analysis of mitochondrial electrical potential. Both synovial fluid (SF) and membrane (SM) cells cultivated after 48 and 144 h did not show differences in the electric potential. The representative histograms obtained by a flow cytometer, show the means of populations with high electric potential (MI) and low mitochondrial electrical potential (M2). In relation to the distribution of the mitochondrial number per cell, data obtained from both cultures after 48 and 144 h showed that there were a significant number of mitochondria per cell, however, without significant differences. Acquisition and analysis were performed using the WinMDI program

The synovial fluid and membrane cells were subjected to an analysis of the mitochondrial membrane potential by Rhodamine 123 fluorochrome. After the acquisition and analysis using the WinMDI program, both synovial fluid and membrane cells cultivated after 48 and 144 h did not show differences in the electric potential. The representative histograms obtained by a flow cytometer, and the means of populations with high electric potential (MI) and low mitochondrial electrical potential (M2) are shown on Fig. 7. The distribution of the mitochondrial number per cell was also analyzed by considering the median fluorescence intensity per cell. The dotplots of both synovial fluid and membrane cells are shown on Fig. 6. Data obtained from both cultures after 48 and 144 h showed that there were a significant number of mitochondria per cell, however, without significant differences (Fig. 7).

## Discussion

Damage in the articular cartilage requires therapeutics innovations that can repair the structure of this tissue in order to offer an efficient treatment for patients. Currently, cell therapy is an essential tool and an important alternative for regenerative medicine. Advances in the development of cell culture allow the development of a variety of culture mediums supplemented with growth factors, amino acids, and several other substances. The best results to cultivate the cells obtained from the synovial fluid and membrane were obtained using MEM (Minimum Essential Medium) and DMEM-H (Dulbecco’s Modified Essential Medium High Glucose), which were effective in maintaining the cells in an undifferentiated and proliferative stage with satisfactory characteristics of growth as previously discussed in the literature [6, 7, 13]. After expansion, freezing, and culture, synovial fluid- and membrane-derived cells were extended to the 11th passage. But, the cells in the 7th passage were analyzed in relation to the morphological and functional aspects. Human adult cells were able to retain a linear growth curve for at least 30 replications [6]. As we demonstrated in our results, though we studied the kinetics of different cells in the 7th passage, the authors also obtained a sigmoidal growth curve. According to De Bari et al. [1] the multipotent capacity of the cells is not influenced by donor age, cell passage and cryopreservation. In addition, they described these cells as showing a limited senescence and able to be expanded to large numbers of cultures. This study demonstrated that human cells maintained their proliferative capacity after the 10th passage. However, in our study we were able to cultivate the equine cells with satisfactory growth and proliferation until the 11th passage. In addition, was demonstrated that synovial fluid-derived MSCs isolated from diseased joints proliferated to >1 × 10^6^ cells at passage 3 and 1 × 10^7^ cells at passage 4 [10]. It has been shown that the synovial tissue can be obtained from different locations on the knee joint [14]. However, it is known that cells derived from the medial limb have more ability to form colonies than those collected from other regions [13]. According to this, we obtained samples of the synovial membrane and fluid from the tibiotarsal and metacarpophalangeal joints. As stated by Koga et al. [15], synovial fluid cells have higher chondrogenic potential, since they have a high production of UDPGD, an enzyme necessary for the production of HA, and are not expressed by other types of stem cells. Besides that, elevated expression of specific markers for chondrogenesis on horse synovial fluid-derived MSCs, such as Sox-9, Col-II, and aggrecan were observed by RT-PCR, as well as cartilage-specific molecules such as COMP in the extracellular matrix [10]. It is known that phenotypic characteristics, such as the production of collagen type II, aggrecan and potential for osteogenic, adipogenic, and chondrogenic differentiation is shared between stem cells obtained from the synovial membrane and fluid [15]. *In vitro* differentiation of horse synovial fluid-derived MSCs demonstrated the multipotency of these cells in osteogenic, adipogenic, chondrogenic, and tenogenic differentiation [10]. In our study, both synovial fluid- and membrane-derived cells demonstrated similar characteristics during the culture and expression of cell markers. Usually, the human mesenchymal stem cells are characterized by the expression of CD105, CD90, Stro-1, and CD73. In addition, this cell type is negative for CD45, CD34, and CD11 [16–18]. Moreover, Kolf et al. [19] stated that the co-expression of STRO-1, CD73 and CD106 is the most efficient combination of markers for mesenchymal stem cells. Krawetz et al. [20] demonstrated that human synovial fluid-derived cells from normal, healthy joints, and from joints with osteoarthritis expressed CD105, CD73 (specific for mesenchymal stem cells) and CD44 (a receptor for hyaluronic acid that can also interact with collagens and MMPs, and can also be expressed in mesenchymal stem cells). In addition, the cells were negative to CD45RO, CD11, and CD34. Herein, we showed that in equines, both the synovial fluid- and membrane-derived cells expressed CD105, CD90, as well as CD117 and CD133. Previously data [10, 21–23] demonstrated that cells derived from these two tissues have a phenotype similar to the bone marrow stem cells, including the expression of CD105, CD90, CD44, and are negative for CD45, CD34 and CD117. In contrast to what is described for horse synovial fluid-derived MSCs [10], our cell lineages had a significant expression of CD45 and CD34, markers that are usually associated to characterize hematopoietic cell lineages. Since, for horses, an immunophenotype for stem cells is not established [16], variations may occur, and it is important to consider the influence of the severity and the acute our chronic stage of the osteochondrosis in the expression of these markers. The expression of CD117, also suggests the hematopoietic capacity of this cell population, as well as the VEGF-R1 receptor being an important mediator in the recruitment, mobilization, angiogenesis and inflammation of cells with a pluripotent potential. Krawetz et al. [20] showed that CD90^+^ cells have increased chondrogenic potential when compared to the CD90^−^ cells. As demonstrated by Nagase et al. [13], CD90 is expressed in 40–60 % of the cells during the first 7 days in culture followed by a gradually decreased expression. Interestingly, the chondrogenic potential also decreased, suggesting that CD90 may be an important indicator of chondrogenic differentiation. The significant difference between the expression of CD90 (≅38 %) in our results in comparison to the literature (>90 %) [10] may be explained by the difference in the cell passages used in both studies, passage 7 in our experiments in contrast to passage 4 used by Murata et al. [10]. Other toti- and multipotency markers, OCT3/4, NANOG, and TRA-1-81, significantly expressed in our cell population, showed no difference between the synovial fluid and membrane. Although similar regulatory mechanism has been suggested for Oct4 in both embryonic and mesenchymal stem cells [24], so far, little is known about the functional role of the pluripotency markers, such as Oct4 and Nanog in adult stem cells [25]. However, the synovial fluid and membrane appear to be a source of stem cells with pluripotency potential, as previously demonstrated [10]. Lee et al. [9] developed a gel composed of collagen type I, hyaluronic acid, and fibrinogen, that encapsulates the cells derived from the membrane to be used to repair the articular cartilage in rats. In addition, the authors stated that direct cell transplantation without a matrix could induce a fibrosis formation. According to Jones et al. [26] there is no difference in chondrogenic cells derived from healthy joints, compared to osteoarthritis and rheumatoid arthritis. Nevertheless, there is an increase in number of cells with proliferative potential in joints with osteoarthritis due to cell communication and release of growth and proliferative factors. This fact was confirmed demonstrating that cells from individuals with osteoarthritis are 20 times more numerous than those obtained from healthy patients. Morito et al. [27] demonstrated that stem cells are increased in the synovial fluid of human beings with joint disease and injury. In that regard, it was demonstrated for horses that the number of colonies of synovial fluid-derived MSCs from diseased joints at passage 0 was significantly increased, when compared to normal joints, suggesting that synovial fluid-derived MSCs could play an important role in the process of degradation, repair, and regeneration of damaged cartilage [10]. Inflammation, which initiates in response to tissue injury, is complex and highly regulated. Once activated, there is a sequential release of mediators which results in the development of classic signs of inflammation: vasodilatation, increased blood flow, higher temperature, and activation of sensory pain [28]. Inflammatory cells are attracted to the area by activating the expression of adhesion molecules [29]. For the first time, it was investigated the expression of specific markers involved in the inflammatory process on cells derived from these tissues in horses. Our results showed that there was a lower expression of the TNF-R1 receptor in the synovial-derived cells, as well as of the chemotactic protein MCP-1, when compared to synovial membrane-derived cells. The other markers analyzed (COX-2, CD11 and CD1a) did not differ significantly in the cell cultures, which can also justify the origin of the persistent inflammatory joint. A decrease in the expression of TNF-R1 was observed during the treatment of joints with goat cells derived from the bone marrow after induced trauma [30]. According to Kobayashi et al. [31] the TNF-R1 induced the proliferation of synoviocytes from patients with osteoarthritis. The expression and role of COX-2 were analyzed in a model of acute arthritis [29], showing that this marker correlates to the signs of the disease. We also showed the expression of that in our cells, but without differences between the synovial fluid- and membrane-derived cells. The MCP-1 is expressed in individuals with disorders in the temporomandibular joint and the values were higher in patients with pain [32]. In addition, recently, it was showed that horse synovial fluid-derived MSCs were strongly positive for MHC class I and moderately for MHC class II [10]. Synovial fluid and membrane cultures showed a significant increase in the expression of Ki67, which is involved in proliferation. It is expressed in phases G1, S, G2, and M. There was a significant decrease in the population of cells in G2/M, which confirms the results of the kinetics of cell growth and viability, where the synovial membrane maintained in culture was significantly reduced, including its proliferative capacity after 144 h of culture. The increased expression of Ki67 was demonstrated also on cells obtained from human osteochondroma [33]. The chaperone HSP-47, responsible for the synthesis of collagen-remodeling proteins and chondrogenic activity, was expressed in both cell types. This heat shock protein is essential for the organization of cartilage and normal endochondral bone formation [34]. However, our data showed that the expression of HSP-47 was significantly higher in synovial fluid-derived cells, indicating that these cells are more efficient for collagen maturation and chondrogenic activity than replicating cell death. Izquierdo et al. [35] studied the expression of HSP-47 in cells obtained from human rheumatoid arthritis, showing that its expression can suggest the stage of the disease. Finally, damage of mitochondrial genome causes mutations or deletions of mitochondrial gene products, leading to an increase in the concentration of reduced respiratory chain intermediates, resulting in the formation of free radicals by self-oxidation. A vicious cycle is proposed: mitochondrial DNA damage affects the respiratory chain function, leading to generation of more free radicals which, in turn, will lead to further damage to mitochondrial DNA [36]. Our results showed a significant amount of mitochondria distributed throughout the cell lines. The presence of active mitochondria in the synovial fluid and membrane-derived cells confirms their high proliferative capacity.

## Conclusion

In conclusion, we confirmed the mesenchymal nature of the synovial fluid- and membrane-derived stem cells by the expression of specific markers as well as the capacity of both cell lineages to be used as a new strategy for cell therapy, especially to treat joint pathologies in order to repair cartilage defects or to be used in articular regeneration with a minimal invasiveness process.
